# Supplementary material for: LZER0: A Cost-Effective Multi-Purpose GNSS Platform
Source: Sensors (Basel). 2022 Oct 29;22(21):8314. doi: 10.3390/s22218314 (PMC9657188; doi:10.3390/s22218314)
Supplement: Supplementary file 1 [file sensors-22-08314-s001.zip › sensors-1970880-supplementary.pdf]

Supplementary Material

LZER0: A Cost-Effective Multi-Purpose GNSS Platform

David Zuliani [1,*], Lavinia Tunini [1], Marco Severin [2], Michele Bertoni [1], Cristian Ponton [1] and Stefano Parolai [1]

[1] National Institute of Oceanography and Applied Geophysics—OGS, 33100 Udine, Italy
[2] SoluTOP SAS, 33050 Pavia di Udine, Italy
* Correspondence: dzuliani@ogs.it

Section SA. This section of supplementary information contains a detailed description of the software system of the LZER0 base platform

As mentioned in the main text, we wrote the scripts for handling the RTKLIB packages installed internally on LZER0 (node side) and the scripts used to display the real-time position time-series of the various remote nodes on a local server (server side/user side) to make them available to the end user via a public web server (see Figures 1 and 2 of the main manuscript).

Internal Software (node side)

The software node runs on the Raspberry Pi SBC, and it consists of:

- RTKLIB software. Even though the entire RTKLIB package is compiled and fully available on the Raspberry Pi, we only used the following Command User Interface Application Programs (see detailed description in the RTKLIB manual available at: https://www.rtklib.com/prog/manual_2.4.2.pdf):
  - str2str
  - rtkrcv
  - rnx2rtkp

Teqc software ([1]) developed by UNAVCO (no longer supported, but still working) and used to translate raw data coming from the GNSS motherboard into RINEX format;

tcsh scripts we wrote ourselves to process data streams coming from the GNSS card connected to the Raspberry Pi via the serial or USB ports, and to run RTK or post-processing engines provided by RTKLIB. Below is a list of shell scripts with their function:

- pi.SetDateTime is started on reboot. It is used to force the operating system date and time, requires an Internet connection, and uses ntpdate (a Linux command) to retrieve and set the current date and time from remote NTP servers. This is necessary because rtkrcv relies on the system's date and time for its operations. If GNSS data and system date and time do not match, rtkrcv cannot operate correctly;
- startup.mount.storage is used to mount an external USB stick after reboot. Since we decided to add an external USB stick, this script is needed to check the integrity of the stick before mounting it. With this code, the operating system can be started even if there is a problem with the USB stick.
- str2str.ublox.tcp is started on reboot. It receives the u-blox raw data stream from a connected GNSS receiver (it can detect whether the GNSS device is connected to the USB port or the serial port of the Raspberry Pi) and redirects the data stream to TCP port 2222. It also translates the raw data stream into RTCM 3.1 format and forwards it to the 3333 TCP port. The streams from 2222 and 3333 are later used by other scripts;
- rtkrcv.ublox.ogs.vrasp.manual is started on reboot. It is used to start rtkrcv in the background and provide access to the rtkrcv command console via telnet on localhost on TCP port 2950. rtkrcv is a powerful RTKLIB command line application program capable of

quickly processing input files and/or data streams (raw observation data from GNSS receivers) to produce real-time positions of GNSS logging stations. It uses a Real-Time Kinematic (RTK) on-the-fly (OTF) algorithm to resolve the integer ambiguity. The integer ambiguity N is one of the most important values to be evaluated during GNSS processing. N is the number of carrier wavelengths that lie between the receiver and a GNSS satellite. OTF is a fast method to determine this value. During the search, N is a float value (FLOAT status), and only if it is evaluated as an integer value (FIX status) can the solution be considered ([2]). Inside the rtkrcv.ublox.ogs.vrasp.manual script, a default configuration rtkrcv.curr.conf is passed to rtkrcv. The configuration file contains dozens of settings for rtkrcv, which cannot be described in this article, as they are well described in the RTKLIB manual (see: https://github.com/tomojitakasu/RTKLIB). We just want to remind that the rtkrcv configuration script contains the data source links (which are used as input streams):

  - generated by str2str.ublox.tcp at TCP port 2222 (or 3333);
  - acquired from external sources as GNSS differential corrections, e.g., from a Networked Transport of RTCM via Internet Protocol (NTRIP CASTER), a server used to forward data streams from GNSS reference base stations to end users on the Internet using the HTTP protocol;
  - and the outputs assigned to the TCP ports:
  - 5754: contains the station coordinates latitude, longitude, and ellipsoidal elevation in the LLH RTKLIB format (plain text); this is also called POS format;
  - 5755: standard National Marine Electronics Association (NMEA) format (see also at https://www.nmea.org/);

  In addition, rtkrcv.ublox.ogs.vrasp.manual records data from the external GNSS reference station (the Master). The data can later be used in post-processing mode together with the data coming from the LZER0 device itself, e.g., with lzer0.pp directly on the LZER0 device or on a remote server from which the data can be downloaded and also further processed with software other than RTKLIB (e.g., GAMIT/GLOBK, [3]).

- lzer0.socatMerge is started on reboot. This script merges the streams available on TCP port 5754 (RTCM format) and 5755 (POS format) into a single output stream on port 9999 (it uses a support TCP port that is 8888). The script is especially important for cadastral LZER0 to communicate over WiFi with commercial programs like SmartRTK. The latter needs information in both streams coming from TCP ports 5754 and 5755;
- str2str.dump.hourly.raw captures the raw data stream generated by the receiver over TCP port 2222 and writes it to a file for backup purposes (the session file duration is set to one hour). The files are stored in hourly directories, which are usually located on the external drive mounted with startup.mount.storage;
- str2str.dump.hourly.pos captures the real-time POS stream generated by rtkrcv on TCP port 5754 and writes it to a file for backup purposes (the session file duration is set to one hour). The files are written hourly to directories, which are usually placed on the external drive mounted with startup.mount.storage;
- compress.gnss.hourly is started every hour. It is used to create a backup (as bz2) of the last hourly logged session;
- build.gnss.pos.daily is started once a day. It creates a single daily POS file, starting from the hourly ones created by str2str.dump.hourly.pos;
- lzer0.pp is started every hour. It is used to run rtkpos in the background. rtkpos is a powerful RTKLIB command line application program that can post-process the input file (raw data from GNSS receivers) and determine the positions of GNSS logging stations. This is the post-processing version of rtkrcv and works with files instead of real-time data streams. We have also implemented this solution because it is generally more robust than real-time techniques (see [4]). Thanks to this approach, LZER0 is able to provide an hourly position with post-processed data, which is a reasonable schedule for structural monitoring systems.

External Software (server side/user side)

Although the processing of the GNSS data providing the positions of the acquisition stations is done on the stations themselves, it is useful to have tools available on the server side to display these data. For

this reason, a web platform was created using node.js, JavaScript, PHP and HTML languages, and the QGIS software.

In the following, we present a list of the main scripts used for the real-time web page for monitoring a network with a GNSS reference station (Master) and one or more GNSS measurement stations (Rovers). The Master station is placed in a stable area, while the measurement stations are placed near the area to monitor a specific phenomenon (e.g. a landslide).

- lzer0.forEverStart: this is a script shell used to start a node.js code list. Normally, each GNSS site that can generate data for display has its own node.js script. These scripts are described below and the syntax name is index.[SITE].js;
- index.[SITE].js: are site-specific node.js scripts (just replace the tag [SITE] with a specific site name, e.g. BRU2 for a GNSS monitoring station within a specific monitoring network). They listen for an HTTP request on a TCP port (configurable by the network manager) and then initiate a TCP socket to the remote GNSS site to receive the data streams from TCP port 5754 (this is the TCP port designated for the POS data stream, as described in rtkrcv.ublox.ogs.vrasp.manual). At the same time, they execute the HTML script index.[SITE].html, which is intended for displaying the data present in the POS stream;
- index.[SITE].html: these HTML scripts contain HTML code and JavaScript code. The following JavaScript libraries were used:
    - socket.io: the library for real-time communication between the browser and the server. It takes the data from the node.js code index.[SITE].js (e.g. index.BRU2.js) and passes it to the rest of the code for coordinate transformation with the JavaScript library proj4 and plots with the JavaScript library smoothie;
    - proj4: this library is needed to convert the geographic coordinates (latitude in decimal degrees, longitude in decimal degrees) available in the POS stream into Universal Transverse Mercator (UTM) projected coordinates (two plane coordinates, north, and east in meters).
    - smoothie: a JavaScript chart library called "Smoothie Charts" was then used to display the real-time data coming from socket.io and converted by proj4;
    - The code can display on a browser a real-time plot including plane coordinates, ellipsoidal height, tracked GNSS satellite, position solution quality, and type of FIX, of a GNSS site capable of providing a POS stream over a TCP socket.
- index.[NET].html: this is the main web page containing the plots produced by the various index.[SITE].html of the network [NET]. This page also contains an interactive map created with QGIS. QGIS is supported by a large community of developers, and a rich repository of plugins is available for the end user. For this work, we used the qgis2web plugin. It generates the web map from the QGIS project (previously prepared on QGIS) including the Brugnera sites. The final map is based on the Leaflet JavaScript library, which can create interactive geographic maps (WebGIS).

Section SB. This section of supplementary information contains the result of the preliminary comparison between the LZER0 platform and YETITMOVES equipment

We performed a preliminary test regarding the repeatability performances of the equipment installed for the monitoring application in the Brugnera area. We installed in BRU2 site a Monitoring LZER0 and a YETITMOVES GNSS equipment. Also the reference site (BRU1) is set up with the same equipment. In this way we guarantee that the corrections are retrieved from equivalents devices. Unfortunately we are not able to provide details about YETITMOVES software because it is a proprietary algorithm. However, we can provide details about the hardware, which is a receiver produced by YETITMOVES that implements a u-blox M8T receiver and uses the MOBI MBGPS-30 antenna (see at: https://www.yetitmoves.it/wp-content/uploads/2022/09/displayce-en.pdf). As described in the main text, LZER0 works in real-time and is capable of producing positions at rate of 1s, whereas YETITMOVES system works on post-processing and the positions are computed using 30s hourly RINEX data and available on hourly basis. Our comparison takes into account the repeatability of the two systems (Figure S1) and demonstrate that real-time LZER0 position has, as a measure of repeatability, a standard deviation which is, on average, 1.7 times the standard deviation of the YETITMOVES equipment (which works in post-processing mode).

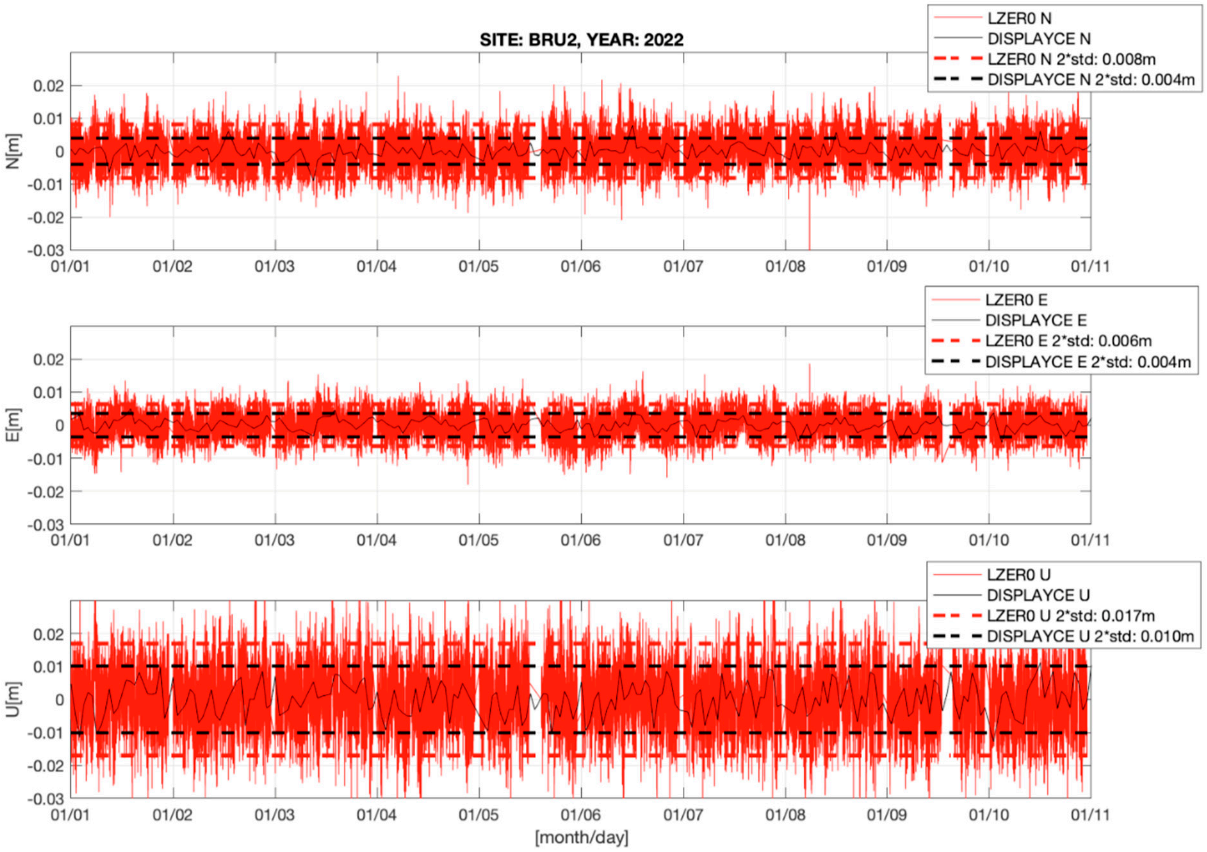

Figure S1. illustrates 10-days time series, generated by LZER0 and YETITMOVES equipment, of the BRU2 positions (North, East and Up components) and their two-sigma (where one-sigma is the standard deviation). LZER0 position is elaborated in real-time at a data rate of 1s, YETITMOVES position is elaborated in post-processing at a data rate of 1 hour. The comparison shows that the real-time LZER0 positions have a standard deviation which are 2 (for the North component), 1.5 (for the East component) and 1.7 (for the Up component) times the post-processed YETITMOVES standard deviations. In any case the horizontal positions repeatability of LZER0 (taking into account a 2 sigma constrain) are of +/- 8mm for the North component, +/- 6mm for the East component and +/-16mm for the Up component.

Section SC. This section of supplementary information contains images of the hardware elements used to realize the LZER0 equipment

This part has been added to better depict every single hardware element listed in section 2.2, the images represent every element by itself without mixing it with other elements or cables (see from Figure S2 to Figure S7b).

Concerning the antenna models selected we would like to notice that the selection was made first looking at some very low cost antennas: ARKNAV A-130 (features available on http://www.arknavgps.com.tw/product/A-130.html), GAACZ-A (features available at https://www.woutersenwouters.be/products/active-gps-antenna-gaacz), ANT-1575R (features available at https://www.datasheetarchive.com/GPS%20Antenna%201575-r-datasheet.html). These antennas are not provided with robust interference rejection systems (usually because of the filter implemented in their Low Noise Amplifier LNA), hence field tests gave intermittent and generally unstable behavior in combination with the RTKLIB software. Therefore we focused on more expensive antennas (see Figure S5) TW3742 (features available at https://www.tallysman.com/product/tw3742-single-band-gnss-antenna-pre-filtered/) and TW4721 (features available at https://www.tallysman.com/product/tw4721-single-band-gnss-antenna/), which implements more performing LNA capable of rejecting the interference produced by other electromagnetic systems such as Long Term Evolution (LTE) and WiFi. In this way we obtained higher stability in the tracking of satellites. The choice made is a compromise between cost, performance and dimensions (the latter is especially important in the Cadastral LZER0 where the designed box has modest dimensions). Unfortunately for low-cost antennas the calibration is not provided (as also indicated in [5]) and we recognize that this is their weak point especially for the measurement of the vertical component. A solution, to overcome this limits, is to use short base-lines ([6]) and the same antennas on both Rover and Master sites ([7]), which is what we have implemented in the Monitoring LZER0 system. In this way the DD technique would allow a substantial reduction of the phase delay ([7]).

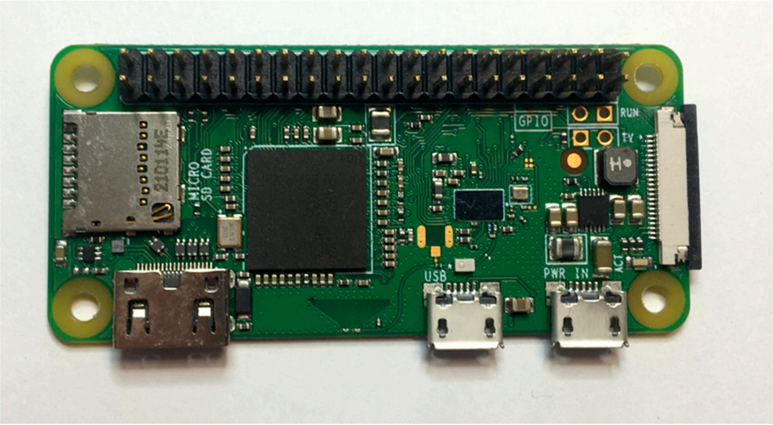

Figure S2. SBC: Raspberry Pi Zero W.

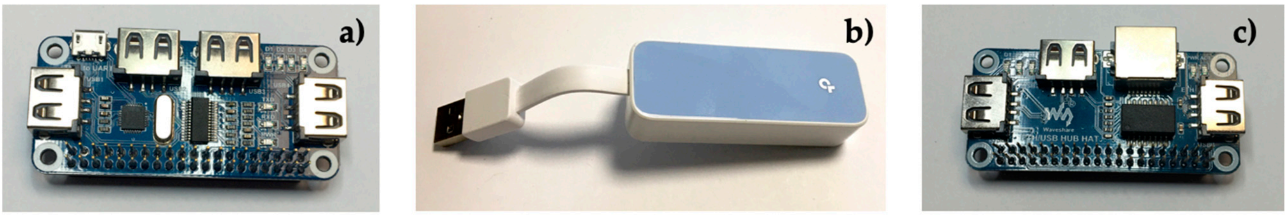

Figure S3. (a) Waveshare USB HUB hat with 4x extended USB 2.0 ports, (b) USB-ethernet adapter tp-link model UE200, (c) Waveshare ETH /USB hub hat.

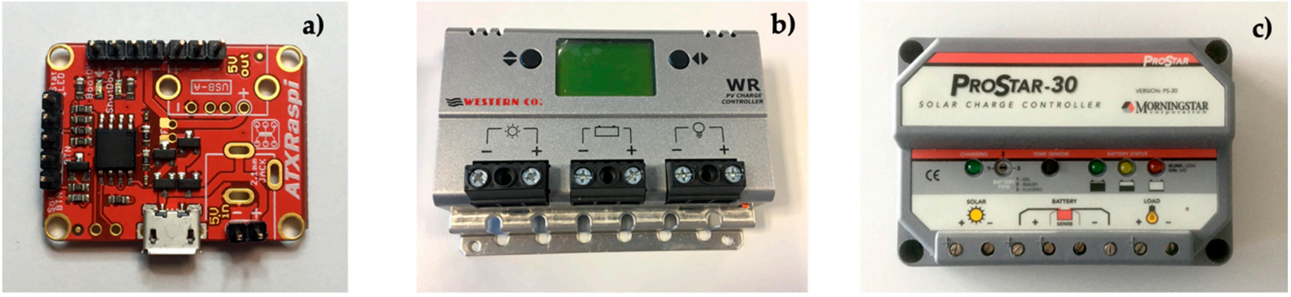

Figure S4. (a) voltage controller and safe shutdown device ATXraspy, (b) voltage controller Western WR30 for battery and solar panel management, (c) voltage controller Morningstar ProStar-30 (an al-ternative to the Western WR30).

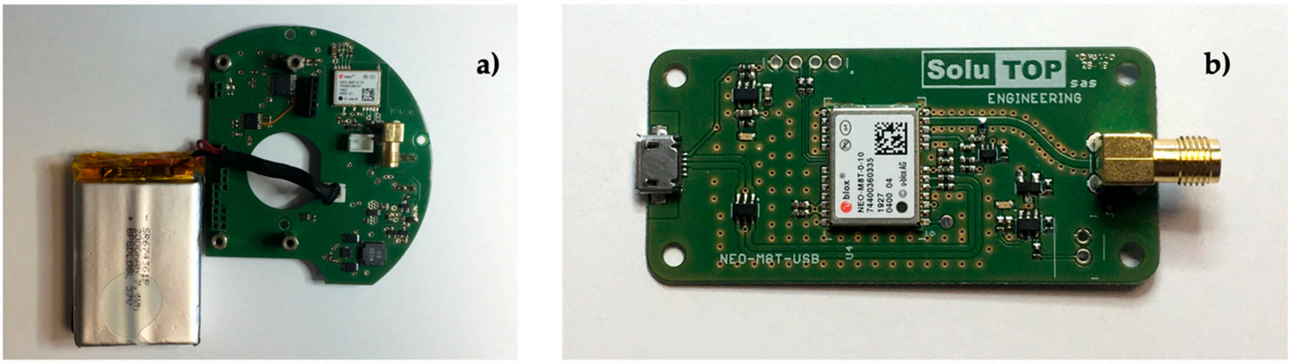

Figure S5. (a) Cadastral LZER0 main board with its 2000mAh LiPo battery, (b) Automotive and Monitoring LZER0 USB board.

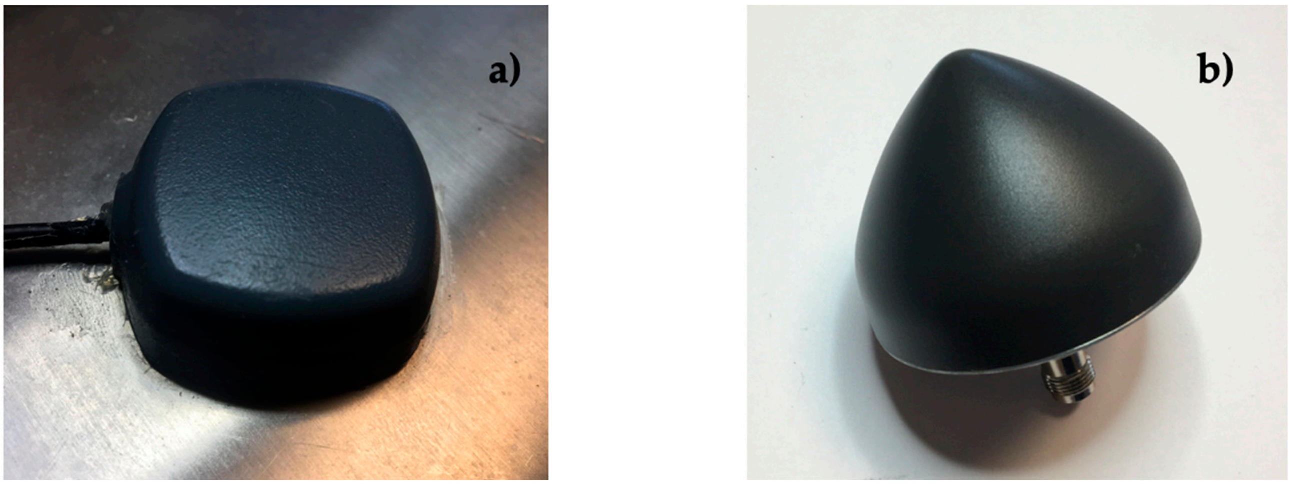

Figure S6. (a) Tallysman TW4721 single-band GNSS antenna used in the Cadastral LZER0 version, (b) Tal-lysman TW3742 pre-filtered single-band GNSS antenna for the Automotive and Monitoring LZER0 models.

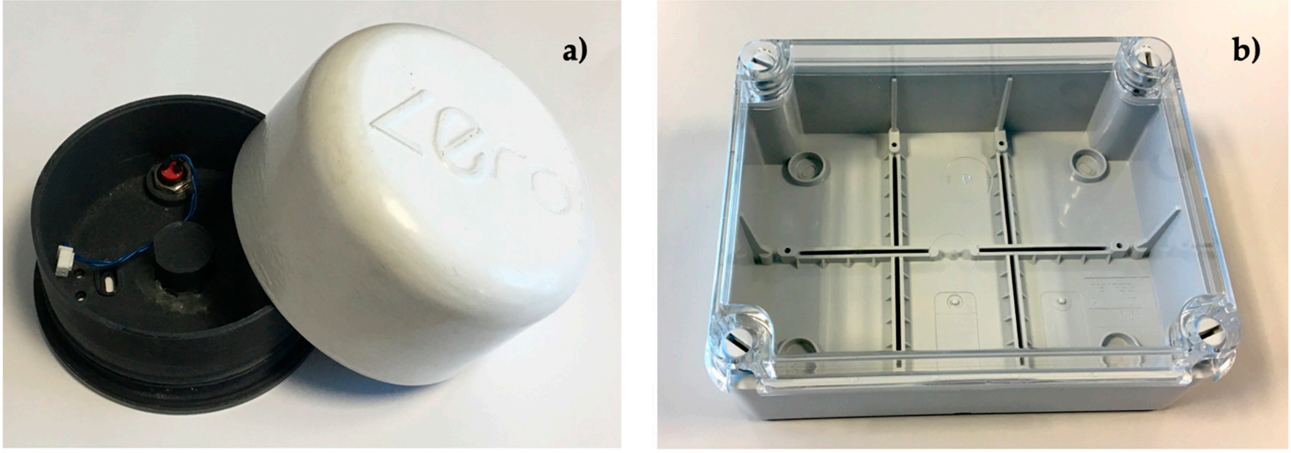

Figure S7. (a) case for Cadastral LZER0 printed with a 3D printer (b) standard Gewiss GW44427 case for Monitoring and Automotive LZER0.

References

1. Estey, L. H.; Meertens C. M.. TEQC: The Multi-Purpose Toolkit for GPS/GLONASS Data and GPS Solutions, published by John Wiley & Sons, **1999**, Vol. 3, No. 1, pp. 42-49, https://doi.org/10.1007/PL00012778.
2. Teunissen, P.; Verhagen, S. GNSS Ambiguity Resolution: When and How to Fix or not to Fix?. In: Xu, P., Liu, J., Dermanis, A. (eds) VI Hotine-Marussi Symposium on Theoretical and Computational Geodesy. International Association of Geodesy Symposia, **2008**, vol 132. Springer, Berlin, Heidelberg. https://doi.org/10.1007/978-3-540-74584-6_22.
3. Herring, T.A.; King, R.; Floyd, M.A.; McClusky, S.C. GAMIT Reference Manual: GPS Analysis at MIT, Release 10.7; Department of Earth. Tech. Rep.; Massachusetts Institute of Technology: Cambridge, MA, USA, **2018**; Available online: http://geoweb.mit.edu/gg/Intro_GG.pdf (accessed on 1 December 2021).
4. Dardanelli, G.; Maltese, A.; Pipitone, C.; Pisciotta, A.; Lo Brutto, M. NRTK, PPP or Static, That Is the Question. Testing Different Positioning Solutions for GNSS Survey. *Remote Sens*. **2021**, 13, 1406. https://doi.org/10.3390/rs13071406.
5. Romero-Andrade, R.; Trejo-Soto, M.E.; Vega-Ayala, A.; Hernández-Andrade, D.; Vázquez-Ontiveros, J.R.; Sharma, G. Positioning Evaluation of Single and Dual-Frequency Low-Cost GNSS Receivers Signals Using PPP and Static Relative Methods in Urban Areas. Appl. Sci. 2021, 11, 10642. https://doi.org/10.3390/app112210642.
6. O'Keefe K. Single versus multiple: how frequencies make a difference in GNSS receivers, Inside GNSS, Volume 11 Number 4, September/October **2016**;
7. Hofmann-Wellenhof, B.; Lichtenegger, H.; & Collins, J. GPS: Theory and practice. New York: Springer, **2001**.
